# Illegal shooting is now a leading cause of death of birds along power lines in the western USA

**DOI:** 10.1016/j.isci.2023.107274

**Published:** 2023-08-01

**Authors:** Eve C. Thomason, Natalie J.S. Turley, James R. Belthoff, Tara J. Conkling, Todd E. Katzner

**Affiliations:** 1 Department of Biological Sciences, Boise State University, Boise, ID 83725, USA; 2 Raptor Research Center, Boise State University, Boise, ID 83725, USA; 3 Idaho Power Company, Boise, ID 83702, USA; 4 US Geological Survey, Forest and Rangeland Ecosystem Science Center, Boise, ID 83702, USA

**Keywords:** Animals, Electricity

## Abstract

Human actions, both legal and illegal, affect wildlife in many ways. Inaccurate diagnosis of cause of death undermines law enforcement, management, threat assessment, and mitigation. We found 410 dead birds collected along 196 km of power lines in four western USA states during 2019–2022. We necropsied these carcasses to test conventional wisdom suggesting that electrocution is the leading cause of death of birds at electrical infrastructure. Of 175 birds with a known cause of death, 66% died from gunshot. Both raptors and corvids were more likely to die from gunshot than from other causes, along both transmission and distribution lines. Past mitigation to reduce avian deaths along power lines has focused almost exclusively on reducing electrocutions or collisions. Our work suggests that, although electrocution and collision remain important, addressing illegal shooting now may have greater relevance for avian conservation.

## Highlights

• Incorrect diagnosis of cause of death of birds impacts law enforcement and management
• Electrocution is thought to kill majority of dead birds found near power lines
• In surveys in western USA, illegal shooting was the leading cause of avian death
• Addressing illegal shooting now may have great relevance for avian conservation

## Graphical abstract

**Figure:**
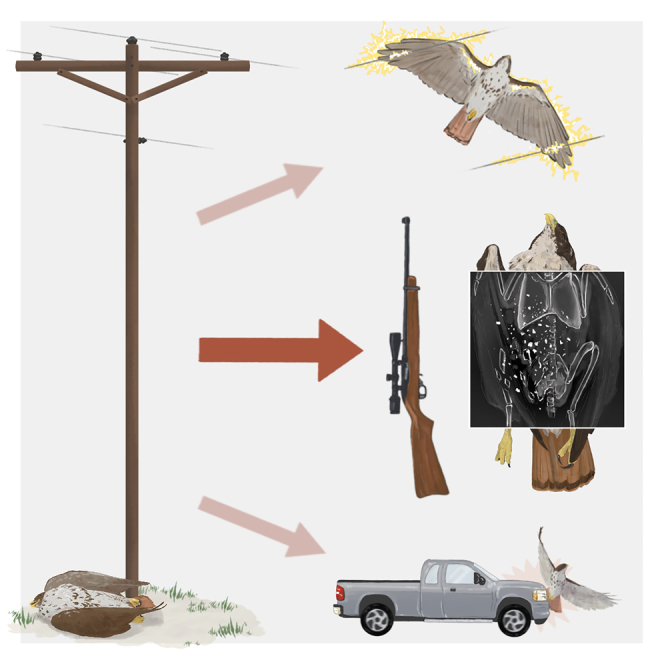

## Introduction

Threats to wildlife are complex and can stem from natural and anthropogenic sources.^1^ Natural mortality factors include intraspecific interactions, starvation, and diseases.^2^^,^^3^ Anthropogenic deaths can result from collisions (e.g., with vehicles, buildings, fences, power lines, wind turbines), electrocution, poisoning, and trapping or shooting.^4^^,^^5^^,^^6^ The killing of wildlife through legal control measures also occurs with regularity and may be demographically relevant.^1^^,^^7^

Power line collisions and electrocutions are well-documented causes of death (CODs) of birds worldwide.^8^^,^^9^ Thus, power utility companies and resource agencies implement conservation strategies to reduce their frequency.^10^ Avian-safe modifications for power lines involve modifying structures and line configurations, insulating energized equipment, installing flight diverters, and installing safe perches and nest platforms.^8^ These modifications can be retroactive, in response to a mortality or outage event, or proactive, as part of electrical reliability programs.^8^

Although avian-safe modifications may reduce electrocutions and collisions, there is evidence that some birds found dead near power lines are killed by other causes. For example, natural history reports suggest that some birds found dead in Utah and Montana had been shot,^11^^,^^12^ and telemetry data suggest that birds also may be poisoned or die from interspecific interactions.^5^ Despite knowledge of the many potential CODs for birds found along power lines, there is a long-standing assumption that most such birds died from electrocution.^13^^,^^14^ Misidentification of COD makes it challenging to evaluate the threats that power lines pose to avian populations and to develop the most efficacious mitigation measures to reduce bird fatalities. Further, understanding how birds die can provide economic benefit to owners of electrical power lines, who expend substantial resources to retrofit infrastructure based on the assumption that reducing the likelihood of electrocution will result in fewer bird deaths. Finally, identifying COD of birds along power lines can help to appropriately assign legal liability and responsibility.

We tested the assumption that electrocution is the most common COD of birds along power lines. We used systematically designed field and necropsy data collection to assess CODs of birds we gathered along power lines across a geographically broad region encompassing four states in the western United States of America (USA) from 2019 to 2022. Our main hypothesis, derived from conventional wisdom, was that birds found dead along power lines died from electrocution. Alternatively, we considered the possibility that birds died from other causes. We tested these hypotheses by evaluating CODs 1) among all carcasses found, 2) between the different types of power lines used to transmit and distribute electricity, and 3) across the different types of protected bird species commonly found dead along power lines.

## Results

**Figure 1:**
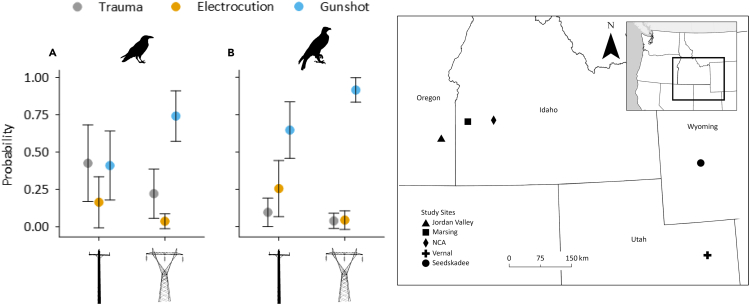
Predicted probability of cause of death of birds found along power lines in the western United States of America Plots show modeled probabilities (±95% CI) of three diagnosed causes of death (trauma, electrocution, gunshot) for (A) corvids and (B) raptors along single-pole distribution lines (on the left of each plot) and multi-pole or lattice transmission lines (on the right of each plot). Modeled probabilities are based on known cause of death of 66 corvids and 101 raptors collected during 2019–2022 at the five study sites shown on the map on the right (Seedskadee National Wildlife Refuge, Wyoming; Vernal, Utah; the Morley Nelson Snake River Birds of Prey National Conservation Area [NCA] in Idaho; Marsing, Idaho; and Jordan Valley, Oregon). Probabilities were calculated separately for each site and averaged for presentation here. Distribution lines are energized to voltages <60 kV, distribute electricity locally, and have wires spaced at smaller distances, making birds more likely to contact two wires at once and die from electrocution. Transmission lines are energized to voltages >60 kV, transmit energy long distances, and generally have larger tower-like support structures, and wires are spaced at greater distances.

We repeatedly surveyed 196 km of power lines (Figure 1) between 2019 and 2022 (see Table S1). Surveys covered 931 poles on distribution lines, 469 on transmission lines, and 72 with underbuild (see Tables S1B, S1C, and Figure 1). We found carcasses of 185 raptors, 132 corvids, and smaller numbers of passerines, waterbirds, Galliformes, or birds that were not identifiable to taxonomic group. The 410 dead birds we found belonged to >48 species (see Table S2). Dead birds were found as fresh (n = 91), decomposed (n = 64), or desiccated (n = 31) carcasses, disarticulated parts (n = 26), bones and feathers (n = 65), only bones (n = 53), or only feathers (n = 80; see Figure S1).

### Overall patterns

Of the 91 fresh carcasses (those that would have been included in previous studies), 53 were shot. Likewise, of all birds in all conditions for which a COD could be identified, the majority (n = 116 or 66%) were shot (see Tables S1D and S2). Shooting occurred at every site and was the most commonly identified COD everywhere except Seedskadee, where similar numbers were shot and electrocuted. The proportion of birds identified as shot ranged from 0.39 (in Seedskadee) to 1.0 (in Marsing and Vernal). Of the remaining birds for which a COD was identified, similar proportions (0.17) died from electrocution and from trauma (see Tables S1D and S2). These two CODs were documented at three of the five survey sites (see Table S1D).

There were 235 birds for which body condition was so poor or evidence so inconclusive that a COD could not be determined. These included species not typically associated with power poles, some of which likely represented prey remains left by predatory birds feeding on the pole (see Table S2).

### Differences among species and between line types

Of the 101 raptors with a known COD, 73 (72%) were shot, 22 (22%) were electrocuted, and six (6%) died from trauma (see Table S2). Of the 66 corvids with a known COD, 42 (64%) were shot, 7 (10%) were electrocuted, and 17 (26%) died from trauma. Eight birds of other taxa died from shooting (n = 1) or trauma (n = 7).

On transmission lines, we identified COD for 89 birds (raptors, corvids, and other). Of these, 72 (81%) were shot, four (4%) were electrocuted, and 13 (15%) died from trauma. On distribution lines, we identified COD for 81 birds, of which 42 (52%) were shot, 24 (30%) were electrocuted, and 15 (18%) died from trauma. On poles with underbuild, we identified COD for 5 birds, of which two (40%) were shot, one (20%) was electrocuted, and two (40%) died from trauma.

Data on COD were best modeled using a multifactor analysis including separate terms for line type and species type; there was weak evidence for an interaction between these two terms (see Table S3). Predicted probabilities suggest that shooting was more likely to be the COD for a bird found dead along transmission than distribution lines (Figures 1A and 1B). That said, both raptors and corvids found dead along power lines of either type generally were more likely to die from gunshot than from other CODs, although for corvids on distribution lines, the probabilities were similar for death by gunshot and trauma. Raptors found on distribution lines were more likely to have died from electrocution than trauma, and corvids found on transmission lines were more likely to have died from trauma than from electrocution. Raptors and corvids had similarly low probabilities of having been electrocuted on both distribution and transmission lines.

## Discussion

In contrast to conventional wisdom, illegal shooting was a prominent, and in most settings, the most common, COD of birds we found along power lines on public lands across a broad swath of the western USA. The assumption that most birds found dead near power lines die from electrocution or collision may once have been broadly correct, perhaps prior to implementation of avian-safe practices along power lines. However, the fact that 66% of the birds for which we identified a COD were shot illustrates the extensive contemporary breadth and frequency of illegal shooting. Beyond its biological relevance, this also has financial implications for utilities and consumers of electricity. This is because the cost-intensive management usually implemented for avian deaths along power lines is almost exclusively focused on mitigating electrocution and collision, while virtually ignoring shooting.

### Trends across types of species and power lines

Shooting was the most likely COD for both raptors and corvids, regardless of whether the carcass was found along a distribution or transmission line. This pattern has substantial implications for design of mitigative actions as, unlike mitigation for electrocution that generally focuses on distribution lines,^8^ corrective action for shooting cannot be focused on one power line type. Rather, these data suggest that addressing shooting will require effort to identify where, when, and why these actions occur, regardless of line type.

Unlike shooting, avian electrocution and trauma are reasonably well understood, and there are good management measures available to mitigate these threats.^10^ The risk of electrocution is thought to be higher on distribution lines than on transmission lines, and our data were generally commensurate with this notion (Figures 1A and 1B;^8^). Raptors and corvids are both relatively large and use electrical infrastructure for perching and nesting,^8^ and regardless of line type, both had similar probabilities of being electrocuted. In contrast, we observed that corvids were more likely than raptors to die from trauma, likely reflecting the frequency with which we found corvid nestlings that had succumbed to trauma sustained from falling from nests.

### Implications for future study

Prior study of avian fatalities along power lines did not always use systematic field surveys, and formal necropsies and radiographs typically only were performed on relatively fresh carcasses of birds (e.g., Lehman et al.^14^). Our data show that limiting necropsies in this way can undermine inference when diagnosing shooting or electrocution. As noted above, many of the dead birds we found were in such poor condition that they would not typically be examined in detail, and thus the prevalence of shooting would have been under-reported if we had not radiographed these poor condition remains or only examined them for burn marks. We do not believe that doing this altered our overall inference about shooting rates because even among birds in reasonable condition, far more died from shooting than from electrocution or trauma.

**Figure 2:**
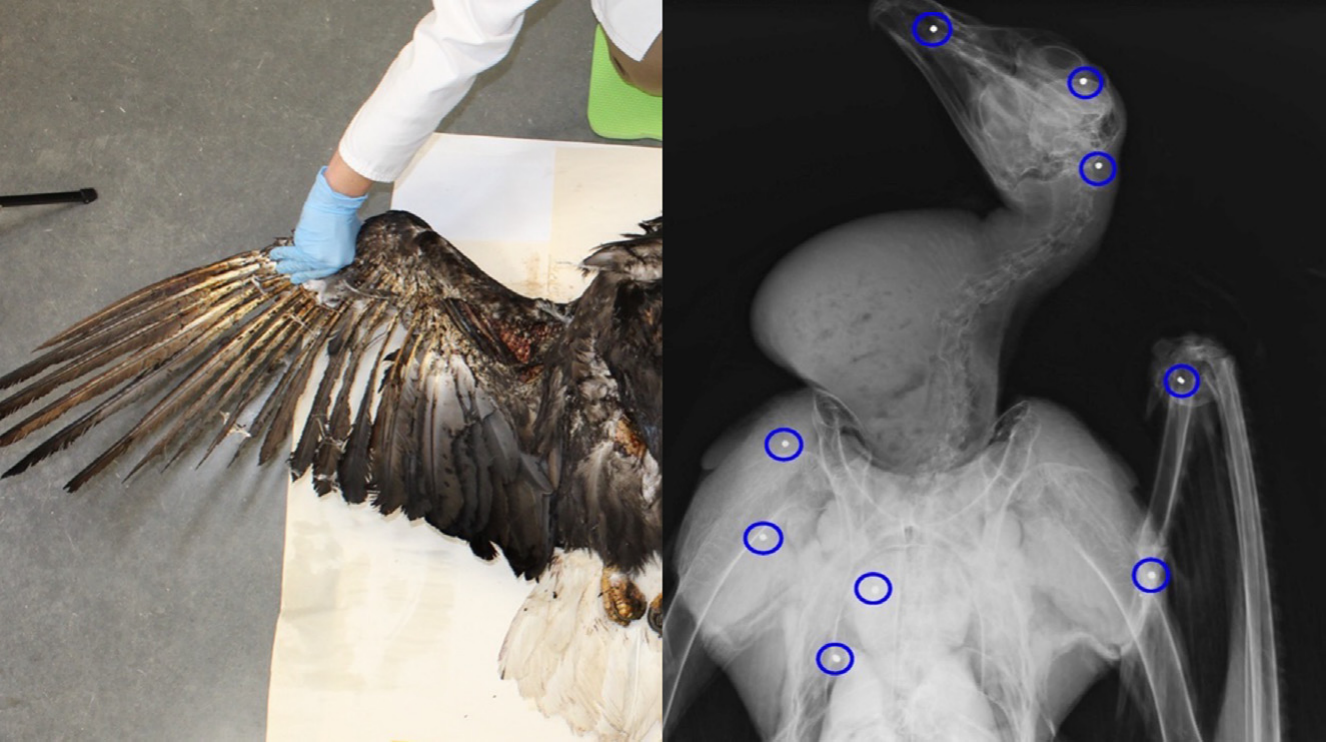
Photograph and radiograph of a bald eagle found dead along a power line near Jordan Valley, Oregon Because of the extensive singeing of the feathers on the wings and body (see photograph), the bird was initially assumed to have died from electrocution. Radiographs of the carcass revealed numerous pellets (in blue circles) in association with fresh wounds, suggesting the bird died from gunshot and then contacted the power lines as it fell to the ground.

Some birds may exhibit external injuries consistent with electrocution, but detailed radiographs can reveal that they also were shot. An example is a bald eagle (*Haliaeetus leucocephalus*) that the power line owner diagnosed from external injury as having died from electrocution (Figure 2). At substantial expense, the utility company spliced lines back together and installed flight diverters to reduce future risk of collision and electrocution. However, our radiographs revealed numerous pellets that were associated with fresh injuries and entrance wounds. These observations suggest that the bird died from gunshot before contacting the power lines as it fell to the ground and thus that the mitigative actions taken were misplaced. Together, these patterns highlight the importance of detailed laboratory examinations, including radiographs, of all avian carcasses when attempting to diagnose possible CODs of birds found along power lines.

### Legal implications & conclusions

Many of the bird carcasses we discovered along power lines were of species that are protected by state and federal laws. As such, the shooting we documented was illegal, and thus our results have substantial implications for wildlife law enforcement. Understanding how wildlife are killed can provide information necessary to plan patrols or launch investigations into criminal activity. In parallel work focused on illegal shooting at the National Conservation Area (NCA), we documented spatial and temporal patterns in the rates at which we found shot birds and we established networks by which this information was channeled to law enforcement agencies.^15^ This information flow was critical to a subsequent successful law enforcement action addressing some of this illegal shooting.^16^ As such, the partnerships we established with utilities, natural resource agencies, and law enforcement illustrate how establishing pathways of communication can promote the immediate application of scientific information toward legal and conservation action.

Illegal shooting of birds along power lines occurs more than previously documented and was common in our study sites on public lands across four states in the western USA. Prior to the development of avian-safe practices, electrocution may well have been the most common COD of large birds along power lines. Our results suggest that, at least in our study areas, this may no longer be the case. Although shooting is known to constrain population growth rates of some species (e.g., golden eagles *Aquila chrysaetos*^5^), it was not previously known to be relevant to so many species across such a large geographic area. Human activity has contributed to the loss of three billion birds across North America.^17^ The insight our work provides into the cause of death of some of these birds can allow power utility companies, resource managers, and law enforcement agencies to develop solutions to manage an anthropogenic threat with widespread impacts.

### Limitations of the study

Although our work shows how surprisingly common is the illegal shooting of birds along power lines, there are knowledge gaps associated with our work that suggest important areas for next steps. One key area for future work might be evaluating the degree to which our results are relevant in other regions or sites where we did not survey. This is important because our results show substantial among-site variability in the frequency of illegal shooting of birds. We expect shooting to be highest in areas with extensive recreational use, similar to our NCA site; future work could test this prediction. Another important area for future work could be to evaluate whether birds are shot in proportion to their abundance on the landscape, or if certain species or types are targeted. For example, there is an active illegal trade in feathers and body parts of eagles that could drive shooting of these species. Another next step could be to utilize rigorously designed surveys using detection rates and carcass removal to evaluate spatial and temporal variation in rates of illegal shooting, as a tool to draw inferences about potential drivers of the activity. Finally, it would be scientifically valuable to understand the demographic significance of this threat relative to the many other threats that birds face. Addressing these knowledge gaps would improve our understanding of this important conservation problem.

## STAR★Methods

### Key resources table

**Table:** 

REAGENT or RESOURCE	SOURCE	IDENTIFIER
**Software and algorithms**		

R, v. 4.2.2	R Core Team^18^	http://www.R-project.org/

### Resource availability

#### Lead contact

Further information and requests for resources should be directed to and will be fulfilled by the lead contact, Eve Thomason (evethomason@u.boisestate.edu).

#### Materials availability

This study did not generate new unique materials.

### Method details

#### Experimental design

We surveyed for bird carcasses along power lines at five study sites in four USA states (Figure 1). Sites were 1) the Morley Nelson Snake River Birds of Prey National Conservation Area in Idaho (hereafter “NCA), 2) near Marsing, Idaho (“Marsing”), 3) in Jordan Valley, Oregon (“Jordan Valley”), 4) northeast Utah near the city of Vernal (“Vernal”), and 5) southwest Wyoming near Seedskadee National Wildlife Refuge (“Seedskadee”). Agriculture, outdoor recreation, and fossil fuel extraction occur throughout our sites. Predominant land cover types are shrub-steppe or native and non-native annual grasslands.^19^ As there are few naturally occurring tall perches or nest sites, birds commonly use power poles for perching and nesting.^14^

Power lines generally are designated as “distribution” (distributing electricity from higher voltage systems to lower voltage end users) or “transmission” (transmitting electricity long distances over high voltage systems; Figure 1). We considered power lines energized to >60 kV as transmission lines and those energized to <60 kV as distribution lines.^8^ Some poles we encountered were classified as having “underbuild” – i.e., they contained both transmission and distribution lines.

We selected specific regions for surveys based on knowledge of where prior surveys had been conducted or on recommendation by power line owners or land managers.^20^ Within each study site we tried to survey power lines in areas with varying human use, shooting pressure, and land cover. All power lines were within 300 m of an accessible road or trail, on public lands managed by the Bureau of Reclamation or the Bureau of Land Management, or within National Conservation Areas or National Wildlife Refuges. We designed surveys to cover similar numbers of transmission and distribution poles at each site, although this was only sometimes possible as both types of power lines were not always present in equal numbers in each region.

#### Field data collection

We searched for bird carcasses in each site at two-to three-week intervals in 2019–2022, although the number of visits to each site varied (see Table S1A). Trained observers walked along each power line route, scanning for avian carcasses within a 20 m belt transect centered on the power line and each power pole. Observers surveyed two power line routes in the NCA by vehicle in 2022.^15^

For each carcass located, we recorded date and location (GPS coordinates), noted species and any visible injuries (e.g., broken bones, singed feathers), assigned a categorical condition (e.g., fresh, decomposed, desiccated, parts, feathers, bones; see Figure S1), took photographs, and collected remains. In some cases, remains were in poor condition and could only be identified to a “bird type,” (e.g., “unknown eagle”, “unknown gull”). We also took photographs of the nearest power pole and recorded utility information such as line type, line configuration, and if avian-safe modifications had been made to the pole. We entered data into “Survey123” forms on camera-enabled handheld Android devices.^21^^,^^22^

Carcasses were delivered to Boise State University (Boise, ID) and placed in a conventional (−20°C) freezer. Ultimately, every carcass, irrespective of condition, underwent a professional necropsy, including radiographs, to help diagnose the cause of death. These examinations were done at the Idaho Department of Fish and Game Wildlife Health and Forensic Lab (WHFL) in Eagle, Idaho and supervised by the lead wildlife health professional at that lab.

#### Laboratory examination protocol

Laboratory examinations to assess cause of death of birds found along power lines were conducted following a standardized procedure. We followed the Idaho Department of Fish and Game Wildlife Health and Forensic Laboratory protocols and other established protocols for necropsy under the supervision of a licensed veterinary pathologist, with modifications specific to this study, as follows.

In most prior studies, only remains in “good condition” were evaluated to identify cause of death (e.g., Lehman et al.^14^). For this study, all remains, regardless of condition, were first examined for external injuries or abnormalities, then all carcasses, bones, and feathers underwent radiographs. If radiographs revealed metal fragments or pellets associated with entrance/exit wounds, broken bones or embedded in major organs, the cause of death was categorized as “gunshot.” Holes through the entire carcass (a “through and through” shot) and feather shearing were also considered indicators of gunshot. As per standard practice, all evidence of shooting was examined for signs of healing to discern whether injuries were fresh (i.e., the bird died from being shot) or healed (i.e., the shooting event did not kill the animal).

If there were broken bones or acute muscle hemorrhaging but no signs of gunshot (as described above) or electrocution (burns or singed feathers), cause of death was categorized as “trauma.” If there was evidence of trauma and also burns or singeing, cause of death was classified as “electrocution”, Although this could artificially inflate the number of cases of electrocution, we felt this was prudent, as evidence of the two together could suggest the bird sustained trauma as it contacted the lines, fell to the ground, or was hit by a vehicle after it contacted energized lines or equipment.

If there were no signs of gunshot or trauma, and there were burns or singed feathers, we categorized the cause of death as “electrocution.” We identified burns and singeing through visual inspection of all remains collected in the field (e.g., carcasses, bones, feathers, feet). Because electrocuted birds can sometimes have very small external burns,^23^ we used magnifying glasses and microscopes during inspection to identify subtle signs of singeing on the undersides of wings and on the lower legs and feet, and we checked internal organs for ruptures of viscera and thermal damage (see below for additional details).

If there was no external or radiograph evidence indicating gunshot, trauma, or electrocution, and the carcass was in suitable condition, we performed full necropsies. Pooled blood in the thoracic cavity and ruptured atrium are indicative of electrocution. Broken bones, lacerations of the liver or other internal organs, or other physical injuries indicate impact trauma. Without any of these or other typical indicators, we listed the cause of death as “unknown.”

Because we considered so many remains that were not examined in prior studies of this type, many bird carcasses were in such poor condition that they could not be given a complete necropsy and we only examined internal organs on the subset of carcasses in which organs were present. Carcasses unsuitable for internal examination included piles of feathers or bones or both, severely desiccated or dismembered carcasses, or carcasses that insects or bacteria had consumed. We only sent samples for disease or toxicant analysis when carcasses were in good condition and cause of death otherwise was not obvious or if there was WHFL interest in results of those analyses. We provide the numbers of carcasses in all conditions in the main text.

### Quantification and statistical analysis

We summarized and analyzed data using R version 4.2.2.^18^ We used a multinomial logit mixed model (package “*mclogit*,” function “mblogit”^24^) to assess the predicted probabilities of the causes of death of birds (our response variable) in response to two categorical predictors – the different types of power lines (distribution and transmission) and the species types (corvids and raptors), with survey site as a random variable. We evaluated model fit for single factor (for either line type or species), multifactor (line type + species), and interactive (line type ∗ species) models using corrected Akaike information criterion (AICc [^25^]). We present results as predicted probabilities with 95% confidence intervals (CI). Small samples sizes for underbuild prevented inclusion of that factor level in model.

## Data Availability

• Some of the data we collected in this work have been, or may be, used in law enforcement actions, and thus cannot be made publicly available. We do certify that any data that can legally be made public will be available to other researchers upon publication of this work, and that confidential data will be made available upon request, as law enforcement agencies allow. • This paper does not report original code. • Any additional information required to reanalyze the data reported in this paper is available from the lead contact upon request.
