# Supplementary material for: Illegal shooting is now a leading cause of death of birds along power lines in the western USA
Source: iScience. 2023 Aug 1;26(8):107274. doi: 10.1016/j.isci.2023.107274 (PMC10440709; doi:10.1016/j.isci.2023.107274)
Supplement: Document S1. Figure S1 and Tables S1–S3 [file mmc1.pdf]

iScience, Volume *26*

Supplemental information

Illegal shooting is now a leading cause of death of birds along power lines in the western USA

Eve C. Thomason, Natalie J.S. Turley, James R. Belthoff, Tara J. Conkling, and Todd E. Katzner

SUPPLEMENTAL INFORMATION

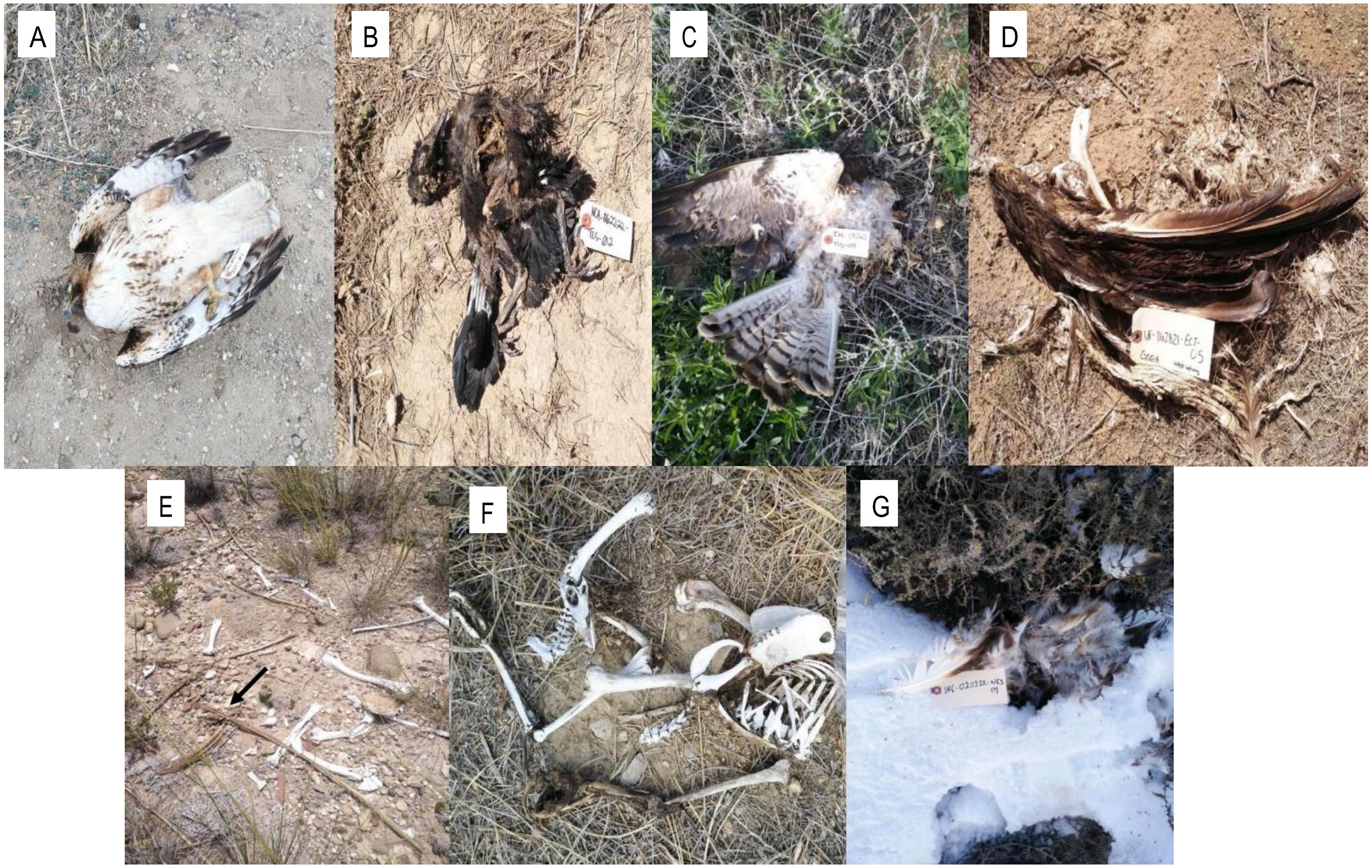

Figure S1. Examples of various bird carcass conditions observed during power line surveys in the western United States of America. Conditions are as follows: A) fresh red-tailed hawk carcass: carcass is intact, no signs of decomposition; B) decomposed common raven carcass: carcass is intact, tissue beginning to decompose, muscle and organs present; C) desiccated Swainson’s hawk carcass: carcass is intact, dry, no muscle or organs present; D) golden eagle parts: only wings or other parts, carcass not intact; E) golden eagle bones and feathers (black arrow is pointing to feathers in the photograph); F) bones of an unknown raptor; G) rough- legged hawk feather spot: >10 feathers.

Table S1. Summary data describing surveys for bird carcasses along power lines at five sites in the western USA. Data include A) survey periods; B) number of poles surveyed; C) distances surveyed; and D) number of avian carcasses documented, organized by cause of death. Survey interval was 2-3 weeks at each site, number in parentheses (in A) refers to number of surveys on each route in that year, and "Visits" (in A, B, and C) refers to total number of times those lines were visited. Surveys in the NCA were on foot or by vehicle, all surveys in other areas were on foot (as described in main text). See Fig. 1 for locations of survey sites and methods used for surveys and to determine cause of death.

A)

Site	State	2019	2020	2021	2022	Visits
NCA (walking)	ID	Feb – Oct (17)		Jan – Dec (26)	Jan – Jul (15)	58
NCA (driving)	ID				Mar – Jul (21)	21
Jordan Valley	OR		Jan – Jun (12)	May – Jul (6)	Jan – Jul (13)	31
Marsing	ID			Jul (1)	Jan – Jul (12)	13
Vernal	UT			May – Jul (3)	May – Jul (3)	6
Seedskadee	WY			May – Jul (3)	May – Jul (3)	6

B)

		Poles*			
Site	State	Dist.	Trans.	Under.	Visits
NCA (walking)	Idaho	226	268	0	58
NCA (driving)	Idaho	57	0	22	21
Jordan Valley	Oregon	210	0	0	31
Marsing	Idaho	0	10	0	13
Vernal	Utah	94	65	0	6
Seedskadee	Wyoming	344	126	50	6
Total		931	469	72	

C)

		Distance (km)*			
Site	State	Dist.	Trans.	Under	Visits
NCA (walking)	Idaho	21	62	0	58
NCA (driving)	Idaho	3	0	2	21
Jordan Valley	Oregon	24	0	0	31
Marsing	Idaho	0	10	0	13
Vernal	Utah	12	15	0	6
Seedskadee	Wyoming	28	14	5	6
Total		88	101	7	

** "Dist." = distribution power lines; "Trans." = transmission power lines; "Under" = poles with both.*

D)

Site	State	Trauma	Electrocution	Gunshot	Unknown	Total
NCA (walking)	Idaho	19	11	64	120	214
NCA (driving)	Idaho	6	3	29	6	44
Jordan Valley	Oregon	2	7	8	56	73
Marsing	Idaho	0	0	6	5	11
Vernal	Utah	0	0	2	10	12
Seedskadee	Wyoming	3	8	7	38	56
Total		30	29	116	235	410

Table S2. Numbers of dead birds found during carcass searches along power lines at five study sites in the western USA, from 2019 through 2022. Study sites were the Morley Nelson Snake River Birds of Prey National Conservation Area near Boise, Idaho, Marsing, Idaho, Jordan Valley, Oregon, northeast Utah near Vernal, and southwest Wyoming near Seedskadee National Wildlife Refuge (see Fig. 1 for a map of these sites). Species we were able to identify are listed in order of the total number of carcasses found and in alphabetical order when numbers are similar; unknowns are listed last. Causes of death are trauma, electrocution ("Electric"), gunshot ("Shot"), unknown ("Unk"). Species types are corvid ("C"), raptor ("R"), and other ("O").

Species	Type	Trauma	Electric	Shot	Unk	Total
common raven *Corvus corax*	C	17	7	42	63	129
red-tailed hawk *Buteo jamaicensis*	R		10	30	17	57
golden eagle *Aquila chrysaetos*	R		8	6	13	27
ferruginous hawk *Buteo regalis*	R	3	1	8	4	16
Swainson's hawk *Buteo swainsoni*	R			12	3	15
rough-legged hawk *Buteo lagopus*	R			10	3	13
horned lark *Eremophila alpestris*	O	2			9	11
mourning dove *Zenaida macroura*	O				10	10
barn owl *Tyto alba*	R	1			5	6
mallard *Anas platyrhnchos*	O				6	6
prairie falcon *Falco mexicanus*	R			3	3	6
bald eagle *Haliaeetus leucocephalus*	R		1	2	1	4
gray partridge *Perdix perdix*	O				4	4
great-horned owl *Bubo virginianus*	R	1			3	4
rock pigeon *Columba livia*	O	1		1	2	4
sage thrasher *Oreoscoptes montanus*	O				3	3
short-eared owl *Asio flammeus*	R				3	3
burrowing owl *Athene cunicularia*	R	1			1	2
great-blue heron *Ardea herodias*	O				2	2
snow goose *Anser caerulescens*	O				2	2
blue-winged teal *Anas discors*	O				1	1
California gull *Larus californicus*	O				1	1
California quail *Callipepla californica*	O				1	1
Canada goose *Branta canadensis*	O				1	1
dark-eyed junco *Junco hyemalis*	O				1	1
European starling *Sturnus vulgaris*	O				1	1
greater sage-grouse *Centrocercus urophasianus*	O	1				1
green-winged teal *Anas carolinensis*	O				1	1
lazuli bunting *Passerina amoena*	O				1	1
loggerhead shrike *Lanius ludovicianus*	O				1	1
long-billed curlew *Numenius americanus*	O				1	1
Nashville warbler *Leiothlypis ruficapilla*	O				1	1
peregrine falcon *Falco peregrinus*	R				1	1

turkey vulture *Cathartes aura*	R			1		1
vesper sparrow *Pooecetes gramineus*	O				1	1
western meadowlark *Sturnella neglecta*	O				1	1
white-crowned sparrow *Zonotrichia leucophrys*	O				1	1
unknown bird	O				24	24
unknown raptor	R		1	1	17	19
unknown eagle	R				10	10
unknown gull	O	2			2	4
unknown corvid	C				3	3
unknown pheasant	O	1			2	3
unknown duck	O				2	2
unknown blackbird	O				1	1
unknown owl	R		1			1
unknown phalarope	O				1	1
unknown sparrow	O				1	1
Total		30	29	116	235	410

Table S3. Top models for generalized linear mixed models to estimate predicted probabilities of the causes of death of raptors and corvids (i.e., "species") on distribution and transmission power lines (i.e., "line type"). Data were collected in the western United States of America from 2019 through 2022. Surveys were within five sites in four states, and we used "site" as a random variable in our models. Cause of death of birds was assessed by radiograph, laboratory examination, and when possible, full necropsy. Of 410 dead birds found, 175 with a known cause of death were included in analyses. Reference categories in the models were "electrocution" for cause of death, "corvids" for species type, and "distribution" for line type.

Model	Delta AICc	AICc weight	Response	Intercept		Line Type		Species Type		Line*Species	
				Estimate	SE	Estimate	SE	Estimate	SE	Estimate	SE
Line + Species + (*Site)*	0	0.62	Trauma	-0.02	0.70	0.88	0.75	-1.91	0.69	-	-
			Shot	0.41	0.61	2.15	0.62	0.04	0.55	-	-
Line * Species + (*Site)*	1.01	0.37	Trauma	0.22	0.69	0.58	1.01	-2.47	0.86	1.32	1.49
			Shot	0.15	0.68	2.46	0.96	0.32	0.68	-0.42	1.24
Line + (*Site)*	10.29	<0.01	Trauma	-1.09	0.53	1.52	0.70	-	-	-	-
			Shot	0.43	0.41	2.15	0.59	-	-	-	-
Species + (*Site)*	13.59	<0.01	Trauma	0.27	0.76	-	-	-2.15	0.76	-	-
			Shot	1.59	0.71	-	-	-0.57	0.51	-	-
